# Maintenance energy requirements and forage intake of purebred vs. crossbred beef cows^1^

**DOI:** 10.1093/tas/txaa008

**Published:** 2020-01-19

**Authors:** Claire E Andresen, Aksel W Wiseman, Adam McGee, Carla Goad, Andrew P Foote, Ryan Reuter, David L Lalman

**Affiliations:** 1 Department of Animal Science, Oklahoma State University, Stillwater, OK; 2 Department of Statistics, Oklahoma State University, Stillwater, OK

**Keywords:** body composition, cow efficiency, feed intake, maintenance, milk yield

## Abstract

The objective of this study was to investigate the impacts of cow breed type and age on maintenance requirements, feed energy utilization, and voluntary forage intake. The main effect of breed type included Angus (ANG; *n* = 32) and Hereford × Angus (HA; *n* = 27) lactating cows. The main effect of age included 2- and 3-yr-old (YOUNG; *n* = 29) and 4- to 8-yr-old (MATURE; *n* = 30) cows. Within breed type and age class, cows were randomly assigned to 1 of 2 pens for a total of 8 pens, each housing 7 to 9 cow/calf pairs. To determine maintenance energy requirements, cows and calves were limit-fed for 105 d to body weight (BW) and body condition score (BCS) stasis. There were no differences between breeds in cow hip height, BW, average milk yield (*P >* 0.31), diet digestibility, or cow maintenance energy requirement (*P =* 0.54). Crossbred cows had greater BCS (*P* < 0.05) throughout the experiment. Efficiency of calf growth was not different between breeds when expressed as feed intake of the cow/calf pair nor as energy intake of the pair per unit of calf BW gain (*P* ≥ 0.31). Young cows produced less milk per day and per unit of BW^0.75^ (*P* < 0.01); however, there was no effect of cow age on maintenance energy requirement, diet digestibility, or efficiency of calf growth (*P* > 0.10). Subsequently, a 45-d experiment was conducted to determine voluntary low-quality forage intake. Cows were housed in dry-lot pens equipped with shade, windbreaks, and feed bunks with free-choice access to clean water and a chopped hay ration was provided ad libitum to determine forage intake. Daily forage intake was lower (*P* = 0.05) for HA compared with ANG (123 vs. 132 g/kg BW^0.75^, respectively) although there was no difference in BW. However, HA cows sustained greater BCS (*P* < 0.01). There was no difference (*P* = 0.60) in forage intake per unit of BW^0.75^ due to cow age. Results indicate similar calf growth efficiency among breed types although crossbred cows maintained greater body energy stores and consumed less low-quality forage during the voluntary intake experiment. These differences could not be attributed to lower maintenance energy requirements. Neither maintenance energy requirement nor calf growth efficiency was different between young and mature cows.

## INTRODUCTION

Biological variation has been reported to exist among cattle breeds for growth, milk production, mature size, and more recently, feed intake (Kuehn and Thallman, 2016; Retallick et al., 2017). Although crossbreeding has historically been used to increase growth (Long et al., 1979a, 1979b), milk yield (Notter et al., 1978; Laster et al., 1979), weaning rate (Wiltbank et al., 1967; Long and Gregory, 1974), and longevity (Cartwright et al., 1964; Spelbring et al., 1977b), further opportunities to capitalize on breed complementarity to reduce feed intake and input costs may exist.

It has been well established that output traits are positively correlated with maintenance requirements of beef cows (Ferrell and Jenkins, 1984b, 1985; Jenkins et al., 1991; NASEM, 2016). Although focused selection on output traits aims to increase performance and revenue, concomitant increases in input traits are most likely occurring. One method to manage this imbalance and better match beef cows to lower input production systems is to use a crossbreeding system that balances breeds with high output with a breed of lower feed intake (Retallick et al., 2017).

Previous literature reported similar maintenance energy requirement between Hereford and Angus cattle (Ferrell and Jenkins, 1985; Taylor et al., 1986; Ferrell and Jenkins, 1987; Solis et al., 1988; Ferrell and Jenkins, 1998). However, substantial change has occurred in these breeds over the past 40 yr. Compared with Angus, Hereford cattle currently average about 19 kg less yearling weight and 9.5 kg less weaning weight due to differences in dam milk production (Kuehn and Thallman, 2016). Furthermore, recent data show that growing Hereford heifers consumed less forage than Angus heifers with no difference in performance (Retallick et al., 2017). Due to a lower genetic potential for growth and milk, Hereford cattle may have lower maintenance requirements and therefore require less feed. In energy-limited environments, this may result in more feed energy available to maintain maternal tissue energy stores and could therefore impact reproductive efficiency (Holloway et al., 1985). Therefore, the objective of this study was to determine maintenance energy requirements, voluntary feed intake, and efficiency of preweaning calf growth for Angus and Hereford × Angus cows.

## MATERIALS AND METHODS

All procedures and protocols were approved by Oklahoma State University Animal Care and Use Committee (#AG-17-26). Experiments were conducted at the Range Cow Research Center near Stillwater, OK. Fall-calving cows and their calves were used in 3 consecutive experiments to evaluate the impact of a crossbreeding program on maintenance requirements, milk production, efficiency of calf growth, diet digestibility, and voluntary feed intake. Treatments included 2 breeding systems: Angus (ANG) or Hereford × Angus (HA), and 2 age classes: 2- and 3-yr-old cows (YOUNG) or 4- to 7-yr-old cows (MATURE). From 4 January 2018 to 20 April 2018, lactating ANG (*n* = 32) and HA (*n* = 27) cows and their calves were used to determine cow maintenance energy requirements and efficiency of calf growth. Calves were sired by 4 Angus (*n* = 42), 3 Hereford (*n* = 7), and 3 Charolais bulls (*n* = 10). All sires ranked within the 30th to the 60th percentile for weaning weight expected progeny difference (EPD). Additionally, an apparent total tract digestibility experiment was conducted using lactating ANG (*n* = 4) and HA (*n* = 4) cows from the same herd. Following weaning, cows were maintained in the same contemporary groups as the maintenance trial and a feed intake trial was conducted from 26 May 2018 to 12 July 2018 to determine voluntary feed intake.

### Maintenance Study

Prior to the initiation of the study, cows were synchronized for timed artificial insemination using a 7-d Co-Synch protocol (Stein et al., 2015). Angus dams were mated randomly to 1 of 2 Angus sires or 1 of 2 Hereford sires. Hereford-sired cows were mated randomly to 1 of the same 2 Angus sires. Following timed artificial insemination, MATURE cows were exposed to Charolais bulls and YOUNG cows were exposed to Angus bulls for 45 d. Bulls were removed and pairs were transferred to the experimental pens during early January. During the first 21 d of the experiment, cows were observed twice daily for standing heat. When estrus was observed, cows were artificially inseminated approximately 12 h after the conclusion of estrus.

Cows and their calves were assigned to experimental pens on 4 January 2018 (day 0). Cows were sorted by breed (ANG and HA) and age (young and mature) and assigned to a pen to achieve a similar average calving date and similar distribution of calf breed of sire. Each breed × age combination had 2 replicates (*n* = 8 total pens). Igenity Beef genomic profile test (Neogen GeneSeek Operations, Lincoln, NE) means for Angus and Hereford × Angus cows are presented in Table 1.

**Table 1.** Genomic profile means for Angus and Hereford × Angus cows

	Breed^1^		Age^2^			*P*-value		
Igenity Beef Profile^4^	ANG	HA	Young	Mature	SEM^3^	Breed	Age	Breed × Age
WW^5^	6.8	6.4	6.5	6.7	0.21	0.19	0.58	0.89
YW^5^	6.8	6.3	6.5	6.5	0.20	0.16	0.90	0.23
ADG^5^	6.4	5.9	6.1	6.1	0.16	0.08	0.93	0.31
RFI^5^	6.5	5.9	6.3	6.0	0.17	0.05	0.25	0.38
Milk^5^	6.14	5.58	6.0	5.7	0.20	0.12	0.36	0.12

^1^ANG = Angus; HA = Hereford × Angus cross; *n* = 4 pens per breed class.

^2^Young = 2 and 3 yr of age, Mature = 4 to 7 yr of age; *n* = 4 pens per age class.

^3^SEM of main effects.

^4^Igenity Beef genomic profile test, Neogen GeneSeek Operations, Lincoln, NE.

^5^WW = weaning weight; YW = yearling weight; ADG = average daily gain; RFI = residual feed intake; Milk = calf weaning weight due to maternal environment and milk.

Experimental pens were equipped with fence-line feed bunks and automatic livestock watering tanks (MiraFount A3465, Miraco Automatic Livestock Waterers, Grinnell, IA). Each pen had a windbreak on both north and south perimeters. Pens provided approximately 154 m^2^ per cow–calf pair, 1.75 linear m of bunk space per cow, and 0.87 linear m of bunk space per calf. Order of feed deliveries were rotated among pens in a clockwise direction every week to minimize any potential confounding effect or order of feed delivery.

A 10-d adaptation period to the experimental ration (Table 2) was used to minimize risk of acidosis. Pairs were supplemented the experimental ration while grazing dormant native pasture for the first 3 d. For the following 7 d, cows and calves were brought into the experimental pens and the amount of ration was increased daily until experimental intakes were achieved. Cows were fed to achieve and maintain body weight (BW) and body condition score (BCS) stasis. Equations from NASEM (2016) were used to estimate dry matter intake (DMI) that would approximate maintenance NE_m_ requirements. Diet protein concentration was formulated to achieve positive degradable protein intake balance and positive metabolizable protein balance (NASEM, 2016).

**Table 2.** Ingredient and nutrient composition of total mixed ration fed during the maintenance and apparent digestibility experiments

	Experiment	
Item	Maintenance	Apparent digestibility
Ingredient, % dry matter		
Hay	33.1	33.1
Corn dried distiller’s grains with solubles	32.4	32.4
Cracked corn	22.7	22.7
Liquid supplement^1^	5.3	5.3
Dry supplement^2^	6.5	6.5
Nutrient composition		
CP, %^3^	19.1	19.1
NDF, %^3^	34.4	32.4
ADF, %^3^	21.1	16.0
Ash, %	8.1	8.6
TDN^4^, %	71.6	73.7
DE^5^, Mcal/kg	3.2	3.3
ME^6^, Mcal/kg	2.6	2.7

^1^Liquid supplement contained 60% DM, 15% CP, 2.0% NaCl, 0.50% P, 0.65% Ca, 70,485 IU/kg Vitamin A, as-fed basis.

^2^Dry supplement contained 42.3% SBM, 33.8% limestone, 8.4% salt, 15.5% sodium bicarbonate, as-fed basis.

^3^CP = crude protein; NDF = neutral detergent fiber; ADF = acid detergent fiber.

^4^TDN = total digestible nutrients. Maintenance experiment TDN was determined from chemical composition and the summative equation using 48 h in vitro digestibility (NRC, 2001). Apparent digestibility-experiment diet TDN was determined using gross energy (GE) digestibility.

^5^DE = digestible energy. Values for the maintenance study were determined as TDN (% DM)/100 × 4.409 (NASEM, 2016). Apparent digestibility-study DE values were determined as (daily GE − daily fecal energy)/kg of DMI.

^6^ME = metabolizable energy. Values determined as DE × 0.82 (NASEM, 2016).

Calves were fed the same diet at the rate of 1.25% of BW. Prior to daily feed delivery, 30% water was added to the ration to mitigate sorting of the ration and to assist with bunk management. For calves, a coccidiostat (Deccox, Zoetis Services, LLC, Florham Park, NJ) was top-dressed at the rate of 0.454 kg/d for the prevention of coccidiosis for the duration of the study.

Feed was offered daily at 0730 hours. In order to separate cow and calf feed intake, calves were penned into a separate creep pen prior to feeding. After cows consumed their feed, approximately 1 h, calves were returned to the pen and had continual access to the creep area. Cows and calves were weighed every other week. Cow feed allotment was adjusted every 14 d in an effort to achieve BW stasis. Daily calf feed allotment was adjusted every other week to provide 1.25% of previous week’s mean BW.

### Milk Yield and Composition

Peak milk yield of cows was measured on d −58. During the trial, milk yield was measured on day 26 and at 28-d intervals thereafter. The procedure described by Wiseman et al. (2019) was used. Cows were milked with a portable milking machine (Portable Vacuum Systems, Springville, UT) and a double separation protocol was employed to allow for the standardization of milk production across all dams. To determine milk composition, a subsample was taken, preserved with 2-bromo-2-nitropropane-1,3-diol and shipped to the Heart of America Dairy Herd Improvement Association laboratory (Manhattan, KS) for chemical analyses.

To adjust for differences in dam-calf separation time, rate of milk production (g/min) was determined by dividing milk yield (g) by separation time (min). Rate of production was then multiplied by 1,440 min to calculate a 24-h milk yield. Milk energy was calculated using the following equation (equations 13–46, NASEM, 2016):

$$\begin{array}{l} E=(0.092\;\times MkFat)+(0.049\times MkSNF)-0.0569 \end{array}$$

where *E* indicates energy content of milk (Mcal/kg), MkFat is milk fat content (%), and MkSNF is milk solids nonfat (SNF) content (%).

### Maintenance Energy Requirements

Maintenance energy requirements were defined as the amount of feed energy that resulted in no net gain or loss of body tissue energy (NASEM, 2016). Maintenance requirements were determined using calculations described by Wiseman et al. (2019) with the following modifications. Efficiency of the mobilization of retained energy (weight loss) was assumed to be 80% of NE_m_ (NASEM, 2016) and the retention of energy (weight gain) was assumed to be 68% of NE_m_ (Freetly, 2019). Cold stress was calculated using equations from NASEM (2016). Therefore, the level of feed intake at which cows maintained BW and BCS (1 to 9) was used to determine energy stasis and calculate maintenance energy requirements.

Cows were weighed and assigned a BCS (1 to 9) by 2 trained technicians at the initiation of the trial and then every 2 wk thereafter. Cows were weighed early in the morning prior to feeding with ad libitum access to water and at least 18 h without feed, therefore, BW taken represented shrunk BW. Body weights and BCS were calculated by fitting a linear regression using the average BW and BCS for each pen at each weigh period against time. Regressed weights were then used to determine average daily gain (ADG) and metabolic midpoint weight. Ultrasonography was conducted on day 75 to determine rib fat (between the 12th and 13th rib) and rump fat.

### Apparent Total Tract Diet Digestibility

In a separate experiment, 4 lactating cows from each breed type were used to determine apparent total tract diet digestibility. Cows used in the digestibility experiment were used in the preceding dry-lot maintenance experiment and were fully adapted to the total mixed ration (TMR). One cow was selected from each pen (*n* = 8) to be representative of each breed and age group from the maintenance trial. Directly after the conclusion of the dry-lot maintenance study the 8 cow–calf pairs were moved to experimental pens. The feeding and sample collection protocol are described in detail by Wiseman et al. (2019). Daily diet samples were collected at 0700 each day. Daily fecal samples comprising 10% of total daily fresh weight were collected. Pooled samples were analyzed for gross energy (GE), fat, acid detergent fiber (ADF), neutral detergent fiber (NDF), and ash content. For feed and feces, GE was determined by bomb calorimetry (Dairy One Forage Laboratory, Ithaca, NY). Fat content was determined utilizing the ether extract method (AOAC, 2012). Both ADF and NDF content were determined according to Van Soest (1991). To determine organic matter (OM) and ash concentrations, samples were ashed in a muffle furnace at 500 °C for 8 h. Digestibility components (GE, OM, NDF, ADF, and fat) were determined as

$$\begin{array}{l} Component\;digestibility=\;\frac{CC_{Feed}-\;CC_{Fecal}}{CC{\;\;\;}_{Feed}}\times 100 \end{array}$$

where CC_Feed_ indicates the concentration of the component in the feed and CC_Fecal_ is the concentration of the component in the fecal matter.

### Voluntary Low-Quality Forage Intake

Following the conclusion of the apparent total tract digestibility trial, pairs were turned out to pasture for 7 d. On 7 May 2018, calves were weaned. At the time of weaning, cows were palpated to determine pregnancy status. A third experiment was initiated on 26 May (day 142) and continued through 12 July (day 189) to determine voluntary low-quality forage intake. Fifty-nine nonlactating, gestating cows were placed in dry-lot pens in their original contemporary groups. This experiment was conducted in the same dry-lot pens as described in the previous experiment. Feed was offered daily at 0700 hours. The diet consisted of a low-quality chopped hay harvested the previous summer from native tall-grass prairie hay meadow. A sugarcane molasses-based supplement (Table 3) was sprayed onto the processed hay and thoroughly mixed. This diet was designed to meet energy and protein requirements sufficient for a nonlactating cow in the second trimester of pregnancy to gain 0.5 kg daily (NASEM, 2016). Prior to feeding each morning, 30% water was added to the feed. Each morning before feed was delivered, feed waste adjacent to the bunks inside the pen (on the concrete feeding pad) and adjacent to the bunks outside of the pen (in the feed alley) was collected and sampled. Every third day, orts remaining in the bunks were collected, weighed, sampled for dry matter (DM) content, and discarded. Weekly feed samples were collected to determine diet DM. Feed samples were dried in a forced air-oven for 72 h to determine diet DM. Samples were then ground through a 1-mm screen and analyzed to determine chemical composition (Dairy One Forage Laboratory, Ithaca, NY). Average feed total digestible nutrients (TDN) and metabolizable energy (ME) available at maintenance feeding level were determined using average chemical composition of the ration and the summative equation for TDN (NRC, 2001).

**Table 3.** Ingredient and nutrient composition of total mixed ration fed to cows during the voluntary feed intake experiment

Item	%, DM basis
Ingredient, % dry matter	
Hay	93.4
Liquid molasses supplement^1^	6.6
Nutrient composition	
CP, %^2^	7.7
NDF, %^2^	61.0
ADF, %^2^	38.8
Ash, %	7.7
TDN^3^, %	55.7

^1^Liquid supplement contained 60% DM, 15% CP, 2.0% NaCl, 0.50% P, 0.65% Ca, 32,000 IU/lb Vitamin A (as-fed basis).

^2^CP = crude protein; NDF = neutral detergent fiber; ADF = acid detergent fiber.

^3^TDN = total digestible nutrients. Values for the voluntary feed intake study were determined from measured chemical composition and the summative equation using 48 h in vitro NDF digestibility (NRC, 2001).

Low-quality forage intake was measured for 47 d. Two consecutive BW measurements were recorded 24 h apart at the beginning and end of the feeding period and a single weight was taken in the middle of the experimental period. Because cattle were fed ad libitum prior to and throughout the experiment, all weights were adjusted to a shrunk BW basis (NASEM, 2016). For each BW recorded, nonpregnant BW was calculated by subtracting the estimated BW of the conceptus (NASEM, 2016). Nonpregnant BW was then used to determine ADG and metabolic midpoint BW. Metabolic midpoint was used to determine feed intake of cows independent of weight differences.

### Statistical Analysis

Data collected for cow performance, maintenance, digestibility, calf performance, and efficiency were analyzed using the MIXED procedure in SAS (SAS 9.4; SAS Institute Inc., Cary, NC). Data were analyzed as a 2 × 2 factorial design with the experimental unit of pen and the fixed effects of breed, age, and their interaction. Milk yield and composition were analyzed as repeated measures using the autoregressive covariance structure to determine the effect of time (month) on milk yield and composition with Satterthwaite approximation to determine the denominator degrees of freedom. For all analyses, treatment means were separated using least square means. Significance was declared if *P* ≤ 0.05 and tendencies were declared at *P* > 0.05 and *P* ≤ 0.10.

## RESULTS

### Cow Performance

Even though feed allocation within each pen was adjusted approximately every 14 d in an effort to achieve pen average weight stasis, some pens had slight weight gain while others experienced minor weight loss. There was no difference in BW between breeds at any point during the experiment (Table 4; *P* ≥ 0.38). As expected, young cows were lighter than mature cows throughout the experiment (*P* < 0.01). Similarly, there was no difference in ADG between age groups (*P* = 0.70). There was a notable breed effect on body composition where crossbred cows began the experiment with a greater BCS (*P* = 0.03) compared with ANG and maintained this difference (*P* = 0.03). Breed differences in BCS were in agreement with ultrasound data indicating crossbred cattle had a tendency for greater rump fat (*P* = 0.06) as well as a numerically greater back fat thickness (*P* = 0.13). There were no differences between breeds in daily feed required to maintain BW, expressed as g/kg BW^0.75^ (*P* = 0.16).

**Table 4.** Effects of breed type and age on feed allowance, body weight, body condition, and pregnancy rates in limit-fed beef cows

	Breed^1^		Age^2^			*P*-value		
Item	ANG	HA	Young	Mature	SEM^3^	Breed	Age	Breed × Age
Feed allowance, g DM/kg BW^0.75^	69.5	65.6	66.5	68.6	1.61	0.16	0.40	0.77
Body weight, kg								
Initial	505	518	485	537	5.76	0.18	<0.01	0.38
Final	516	516	491	540	4.70	0.97	<0.01	0.81
ADG, kg/d	0.13	−0.03	0.07	0.03	0.21	0.21	0.70	0.31
BCS^4^								
Initial	5.09	5.44	5.37	5.16	0.07	0.03	0.12	0.13
Final	5.05	5.32	5.19	5.18	0.05	0.02	0.87	0.04
Carcass ultrasound								
12th rib fat, cm	0.14	0.21	0.20	0.14	0.02	0.13	0.17	0.77
Rump fat, cm	0.16	0.24	0.22	0.17	0.02	0.06	0.24	0.84
Frame								
Hip height, cm	51.7	52.0	51.2	52.5	0.17	0.29	<0.01	0.37
Pregnancy rate, %^5^	94.4	89.3	87.3	96.4	—	0.22	0.06	0.61

^1^ANG = Angus; HA = Hereford × Angus cross; *n* = 4 pens per breed class.

^2^Young = 2 and 3 yr of age, Mature = 4 to 7 yr of age; *n* = 4 pens per age class.

^3^SEM of main effects.

^4^BCS = body condition score (1 to 9; Wagner, 1988).

^5^Cows were synchronized and timed artificial insemination was performed on d −45. Cows were then exposed to fertile bulls for 43 d. Bulls were removed on day 0 and pregnancy was determined via rectal palpation at weaning.

### Diet Digestibility and Feed Energy Concentration

Results from the total tract digestibility experiment are shown in Table 5. Breed groups were fed at the same rate (79.5 g/kg BW^0.75^). There were no differences in OM, GE, NDF, ADF, or fat digestibility between Angus and HA cows (*P* ≥ 0.57). Although mature cows required greater feed intake (*P* = 0.03) to maintain BW, there was no effect of age on OM, GE, NDF, nor ADF digestibility, mature cows had greater fat digestibility than young cows (*P* = 0.05).

**Table 5.** Effects of breed type and age on diet apparent total tract digestibility in limit-fed beef cows

	Breed^1^		Age^2^			*P*-value		
Item	ANG	HA	Young	Mature	SEM^3^	Breed	Age	Breed × Age
DMI, kg	8.6	8.7	8.3	9.0	0.14	0.72	0.03	0.03
OM digestibility, %^4^	73.5	73.5	72.7	74.4	0.98	0.99	0.29	0.44
GE digestibility, %^4^	74.4	75.4	74.1	76.0	0.40	0.63	0.47	0.59
NDF digestibility, %^4^	59.8	61.3	59.0	62.2	1.80	0.57	0.28	0.38
ADF digestibility, %^4^	62.1	60.0	57.7	64.4	5.08	0.78	0.40	0.73
Fat digestibility, %^4,5^	90.0	90.0	89.4	92.6	0.32	0.89	0.05	0.50

^1^ANG = Angus; HA = Hereford × Angus cross; *n* = 4 cows per breed class.

^2^Young = 3 yr of age, Mature = 4 and 5 yr of age; *n* = 4 cows per age class.

^3^SEM of main effects.

^4^OM = organic matter (AOAC, 2012); GE = gross energy via bomb calorimetry; NDF = neutral detergent fiber (Van Soest et al., 1991); ADF = acid detergent fiber (Van Soest et al., 1991).

^5^Fat digestibility determined via ether extract method according to AOAC (2012).

### Milk Production and Composition

**Figure 1.**
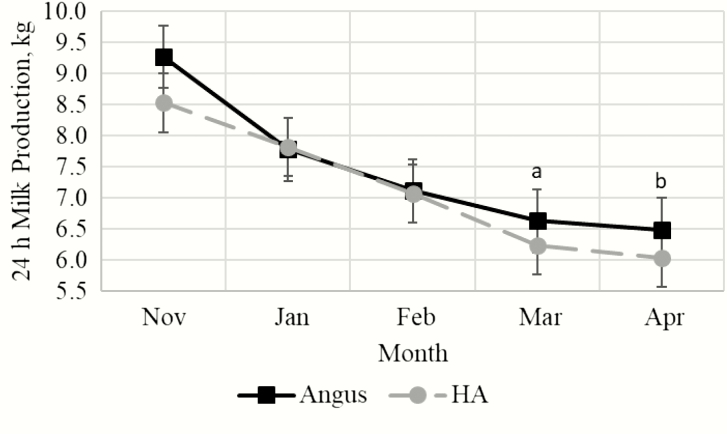
Effect of breed type (ANG = Angus, HA = Hereford × Angus cross) and month on milk yield. ^a^Within month, means tend to differ, *P* = 0.09. ^b^Within month, means differ, *P* = 0.05.

Milk yield was not different for Angus compared with Hereford-sired cows when measured in November during early lactation (Figure 1; *P* = 0.11). Similarly, there were no differences in 24 h milk production when measured during January and February (*P* ≥ 0.90). Angus cows tended to maintain yield persistency during March (*P* = 0.09) with greater (*P* = 0.05) yield during April compared with crossbred cows.

When averaged across the 84-d trial period, milk yield was not different (*P* = 0.11) between breeds (Table 6). Evaluation of milk composition indicated no differences in protein, lactose, SNF, or milk urea nitrogen (MUN) between breeds (*P* > 0.20). However, HA had a greater concentration of milk fat (*P* = 0.03) compared with Angus. This resulted in greater milk energy per kg of milk produced (*P* = 0.03) for HA cows.

**Table 6.** Effects of breed type and age on milk yield and milk composition in limit-fed beef cows during mid- and late-lactation

	Breed^1^		Age^2^			*P*-value		
Item	ANG	HA	Young	Mature	SEM^3^	Breed	Age	Breed × Age
Milk yield, kg/d	7.6	7.1	6.6	8.2	0.19	0.11	<0.01	0.17
Milk energy, Mcal/kg^4^	0.71	0.74	0.72	0.73	0.01	0.03	0.61	0.39
Milk fat, %	3.5	3.8	3.6	3.8	0.07	0.03	0.94	0.52
Milk protein, %	3.1	3.1	3.2	3.1	0.02	0.90	0.03	0.08
Milk lactose, %	4.9	4.9	4.9	4.8	0.02	0.40	<0.01	0.69
Milk SNF, %^5^	9.1	9.1	9.2	9.0	0.02	0.57	<0.01	0.02
Milk MUN, %^5^	14.3	15.2	14.9	14.6	0.46	0.21	0.60	0.56

^1^ANG = Angus; HA = Hereford × Angus cross; *n* = 4 pens per breed class.

^2^Young = 2 and 3 yr of age, Mature = 4 to 7 yr of age; *n* = 4 pens per age class.

^3^SEM of main effects.

^4^Milk energy production (Mcal NE_m_), calculated using NASEM (2016) equation 13–46: (0.092 × % Fat) + (0.049 × % SNF) − 0.0569.

^5^SNF = solids nonfat; MUN = milk urea nitrogen.

There were significant age effects on milk yield and milk composition. Mature cows had significantly greater 24 h milk production (*P* < 0.01) compared with young cows. Although mature cows had greater protein, lactose, and SNF concentration (*P* ≤ 0.05), there were no differences in milk fat or MUN (*P* ≥ 0.66). Although milk composition differed between age groups, there was no difference in milk energy per kg of milk produced (*P* = 0.61).

### Maintenance Energy Requirements

Due to slight weight gain or loss experienced within each pen, equations from NASEM (2016) were used to adjust maintenance requirements for changes in BW and body composition. The amount of energy required for cattle to achieve BW stasis was not different between breeds (*P* = 0.16; Table 7). Equations from NASEM (2016) were used to estimate the daily net energy required for pregnancy. Because cows averaged 60 d pregnant during the 84-d experimental period, the estimated energy partitioned to pregnancy was negligible at 0.2 Mcal/d (data not shown). There was no difference in calculated energy required for cold stress among breeds (dependent primarily on differences in BCS) over the experimental periods (*P* = 0.60). After accounting for energy partitioned to milk production and to maternal tissue energy change (NASEM, 2016), there were no differences in maintenance energy requirements between Angus and HA cows (*P* = 0.68).

**Table 7.** Effect of breed type and age on energy intake and maintenance requirements of beef cows

	Breed^1^		Age^2^			*P*-value		
Item	ANG	HA	Young	Mature	SEM^3^	Breed	Age	Breed × Age
ME intake, Mcal/d^4^	19.0	18.1	17.5	19.5	0.38	0.16	0.02	0.81
Milk energy, Mcal ME/d^5^	8.3	7.9	7.4	8.8	0.45	0.60	0.08	0.47
Tissue retained energy, Mcal ME/d^5^	0.72	−0.22	0.38	0.12	0.41	0.18	0.69	0.32
Cold stress energy, ME/d^6^	0.99	0.75	0.90	0.84	0.30	0.60	0.89	0.81
Maintenance energy^7^								
kcal ME/kg BW^0.75^	93.0	95.7	94.3	94.4	4.31	0.68	0.99	0.96
kcal NE_m_/kg BW^0.75^	60.1	61.9	61.0	61.0	2.79	0.68	0.99	0.96

^1^ANG = Angus; HA = Hereford × Angus cross; *n* = 4 pens per breed class.

^2^Young = 2 and 3 yr of age, Mature = 4 to 7 yr of age; *n* = 4 pens per age class.

^3^SEM of main effects.

^4^ME = metabolizable energy; Mcal = megacalories.

^5^Milk and tissue retained energy was converted to ME basis using equations from NASEM (2016).

^6^Cold stress energy estimated using equations from NASEM (2016).

^7^ME = DE × 0.82 and NE_m_ = 1.37ME − 0.138ME^2^ + 0.0105ME^3^ − 1.12.

Young cows required fewer Mcal per day (*P* = 0.02) for weight stasis and tended to produced less daily milk energy (*P* = 0.08) than mature cows (Table 7). However, there were no differences in maintenance energy requirements between age groups (*P* = 0.99).

### Calf Dry-lot Performance

Calves were limit-fed 1.25% of BW of the same TMR as the dams received. Although not significantly different, HA calves were slightly lighter than ANG calves at the initiation of the experiment (Table 8; *P* = 0.11). This difference in BW resulted in a trend for lower feed amount provided to HA calves (*P* = 0.07). However, there was no difference in ADG (*P* = 0.62) or change in BW (*P* = 0.41) between breeds over the 84-d period. Final weights and 205-d adjusted weaning BW were not different between breeds (*P* ≥ 0.33).

**Table 8.** Effect of breed type and age on calf energy intake, performance, and efficiency of energy use

	Breed^1^		Age^2^			*P*-value		
Item	ANG	AH	Young	Mature	SEM^3^	Breed	Age	Breed × Age
Calf BW, kg								
Initial	165	153	150	169	3.93	0.11	0.03	0.64
Final	255	242	235	262	5.33	0.15	0.03	0.51
Total calf gain	90	88	86	93	1.70	0.45	0.04	0.34
ADG	1.08	1.04	1.01	1.11	0.02	0.22	0.04	0.63
Adjusted WW	240	233	231	242	4.60	0.33	0.15	0.26
Cow energy intake, cumulative Mcal ME^4^	1,594	1,517	1,474	1,638	31.57	0.16	0.02	0.81
Calf energy intake, cumulative Mcal ME^4^								
TMR	432	399	390	442	9.67	0.07	0.02	0.56
Milk	695	665	619	741	37.69	0.60	0.08	0.47
Total	1,127	1,064	1,009	1,183	39.37	0.32	0.04	0.41
Pair cumulative Mcal Feed ME^4^	2,027	1,917	1,864	2,079	30.91	0.07	<0.01	0.95
Calf gain:calf TMR energy intake^5^	209	221	220	210	2.32	0.02	0.04	0.83
Calf gain:calf energy intake^6^	80.1	83.5	85.0	78.7	2.40	0.37	0.14	0.63
Calf gain:pair energy intake^7^	44.6	46.0	45.9	44.7	1.05	0.39	0.45	0.46

^1^ANG = Angus; HA = Hereford × Angus cross; *n* = 4 pens per breed class.

^2^Young = 2 and 3 yr of age, Mature = 4 to 7 yr of age; *n* = 4 pens per age class.

^3^SEM of main effects.

^4^Mcal = megacalorie; ME = metabolizable energy.

^5^Calf BW gain in grams · Mcal of calf TMR intake.

^6^Calf BW gain in grams · Mcal of calf TMR intake and milk intake.

^7^Calf BW gain in grams · Mcal of pair TMR intake.

When evaluating differences between age groups, calves from young cows were lighter at experiment initiation (*P* = 0.03) than calves from mature cows. Calves from mature cows had greater ADG and maintained the BW difference throughout the course of the experiment (*P* ≥ 0.03). However, adjusted weaning BW was not different between age groups (*P* = 0.15).

Feed efficiency ratios were measured as calf BW gain to feed energy intake, calf BW gain to total energy intake (calf TMR energy + milk energy), or calf BW gain to feed energy intake of the cow and calf combined. Calves from HA had improved gain:feed energy ratio (*P* = 0.02). This is consistent with reported genomic scores for residual feed intake (RFI) for this herd where HA cows had lower (more efficient) scores for RFI compared with ANG. Although there was a tendency for HA pairs to consume less energy, there were no breed differences in efficiency of calf growth when evaluated as calf gain to total calf energy intake or calf gain to cow and calf TMR energy intake (*P* ≥ 0.37). Although young cows and their calves consumed less energy than mature pairs (*P* > 0.01), there were no differences in feed efficiency ratios between age groups (*P* ≥ 0.46).

### Voluntary Low-Quality Forage Intake

Cows were fed a low-quality forage for 47 d to determine voluntary forage intake. Throughout the 47-d experiment, there was a tendency for HA cows to weigh more than ANG cows (Table 9; *P* ≤ 0.09). Although there was no difference in ADG over the duration of the intake experiment (*P* = 0.81), there was a tendency for HA cows to weigh more at the end of the experiment (*P* = 0.09). Similar to the maintenance study, HA cows initially had greater BCS (*P* < 0.01) and maintained that difference throughout the experiment resulting in a tendency for greater final BCS (*P* = 0.07). When daily feed intake was expressed as kg/d or g/kg BW^0.75^, HA cows consumed less (*P* ≤ 0.05) than ANG.

**Table 9.** Effect of breed type and age on cow body weight, body condition and voluntary forage intake

	Breed^1^		Age^2^			*P*-value		
Item	ANG	HA	Young	Mature	SEM^3^	Breed	Age	Breed × Age
BW, kg								
Initial	538	548	517	569	2.93	0.07	<0.01	0.39
Final^4^	570	580	551	599	3.06	0.09	<0.01	0.11
BW gain	31.9	31.9	34.3	29.6	1.73	0.99	0.13	0.11
ADG	0.67	0.66	0.72	0.62	0.03	0.81	0.10	0.09
BCS^4^								
Initial	5.60	6.22	5.87	5.94	0.08	<0.01	0.55	0.08
Final	5.77	6.29	5.99	6.07	0.15	0.07	0.72	0.29
Intake								
DMI, kg/hd	15.7	14.8	14.7	15.8	0.24	0.05	0.03	0.09
DMI, g/kg BW^0.75^	131.9	122.6	126.3	128.1	2.23	0.04	0.60	0.13

^1^ANG = Angus; HA = Hereford × Angus cross; *n* = 4 pens per breed.

^2^Young = 2- and 3-yr-old cows, Mature = 4- to 7-yr old cows; *n* = 4 pens per age group.

^3^SEM of main effects.

^4^Initial BW was a covariate.

^5^BCS = body condition score (1 to 9; Wagner, 1988).

Mature cows weighed more than young cows throughout the experiment. Although young cows tended to have a slightly greater ADG (*P* = 0.10) than mature cows, there was no difference in BW change between age groups (*P* = 0.13). Although daily intakes (kg/d) were lower for young cows compared with mature cows, there were no differences in intakes between age groups when expressed as g/kg BW^0.75^.

## DISCUSSION

It is well established that a correlation exists between mature size and DMI in beef cows and beef cow mature BW is an important consideration for feed intake and stocking rate considerations (NASEM, 2016). In the current experiment, there was a tendency for the HA cows to weigh more at the initiation of the voluntary feed intake experiment. However, there was no difference in cow body weight or hip height measured at other times throughout these experiments. The mean mature weight EPD of Hereford sires was 17 kg below breed average and the mean mature weight EPD of Angus sires was 22 kg below breed average (data not shown). For perspective, Hereford sires’ mean mature weight EPD ranked in the 6th percentile lowest for the breed, while Angus sires’ mean mature weight EPD ranked in the 7th percentile lowest for the breed. According to the recent work by Zimmermann (2019), mean mature weight of a large population of industry-current Angus- and Hereford-sired cows did not differ. Therefore, with current study sires having similar percentile ranking within each breed, differences in mature weight would not be expected.

Hereford-sired cows maintained greater BCS throughout these experiments. This could be partially explained by the tendency to produce less milk during early-lactation and lower milk yield persistence during late-lactation. Although overall mean milk yield did not differ between the 2 types, the tendencies for lower early- and late-lactation yield seem to agree with lower weaning BW due to milk reported by Kuehn and Thallman (2016). Other research has shown increased milk yield in crossbred animals due to the effects of heterosis (Cundiff et al., 1974; Montana-Bermudez et al., 1990). Holloway et al. (1985) found that Angus–Hereford females utilized extra nutrients for increased fat thickness as opposed to Angus cattle that had a greater propensity to utilize extra nutrients for increased milk production and thus calf growth. These authors found no difference in milk production between Angus and Angus–Hereford females when consuming fescue-legume pasture. However, with increased nutrient availability in fescue-legume pasture, Angus cattle had increased milk production compared with Angus–Hereford, suggesting that nutrient availability limited expression of milk production in cattle with greater lactation potential.

Although there was a tendency for greater milk fat and overall milk energy concentration in HA cows, there was no difference in daily mean or estimated cumulative milk energy production between the 2 breed types. Researchers (Marston et al., 1992; Brown and Lalman, 2010) have reported that percent milk fat decreases as milk yield increases.

All cows were managed as a contemporary group for 6 mo prior to the beginning of these experiments and were provided access to abundant forage. Under those conditions and as expected, young cows weighed less compared with mature cows throughout the lactation experiment. Similarly, there were no differences in BCS or ultrasound fat depth between age groups at the initiation of the lactation experiment. When evaluating the effect of age on cow condition, Renquist et al. (2006) found that minimum BCS occurred at 3 yr of age. Likewise, Choy et al. (2002) found that BCS increased up to 6 yr of age. The results in the current study are similar to Hudson et al. (2010) who found no difference in BCS between young cows (≤3 yr) and mature cows (≥4 yr). When Banta et al. (2008) evaluated 2-yr-old, 3-yr-old, and mature cows, there was a difference between age classes in initial BCS where 2-yr-olds had the greatest BCS, mature cows had the lowest BCS, and 3-yr-old cows were intermediate. However, in both previously mentioned studies, younger cows lost a greater amount of BCS throughout the production cycle resulting in no differences in prebreeding BCS between age groups (Banta et al., 2008; Hudson et al., 2010). This suggests that while BCS may not differ greatly between age groups, mature cattle may be capable of maintaining condition during physiologically stressful periods (i.e., early lactation, weaning, etc.). Because feed intake was manipulated to achieve weight stasis and because BCS was similar at the initiation of the current lactation experiment, no differences in BCS change would be expected.

Milk production increases linearly to maximum production from first calving to 6 (Neville, 1974) or 8 yr (Rutledge et al., 1971). Lower milk yield in young cows would be beneficial to allow body condition maintenance. In this experiment, young cows produced 19% less milk and 20% less milk energy compared with mature cows. Although Brown and Lalman (2010) found few differences in milk composition based on sire breed, very little data exist evaluating differences in milk composition and quality between ages or parities (NASEM, 2016).

A recent study by De La Torre et al. (2019) found that cows selected for low RFI had increased dry matter and OM apparent digestibility suggesting that efficiency is partially driven by variation in digestive traits. There were no breed type or age differences in apparent total tract digestibility in the current experiment when cows were fed at the same relative amount required to achieve weight stasis during lactation.

Several authors have reported variation in maintenance energy requirements between breed types as a function of differences in milk production and mature size (Ferrell and Jenkins, 1984a, 1984b; Solis et al., 1988; NASEM, 2016). Because overall BW, hip height, and overall milk energy yield did not differ between breed types in this experiment, perhaps it is not surprising that estimated maintenance energy requirements were not different. However, Ferrell and Jenkins (1987) and Solis et al. (1988) found that maintenance energy requirements of Angus × Hereford cross cattle were less than both the parent breeds.

Maintenance requirements did not differ between young and mature cows in this experiment. Neville (1971) reported no change in maintenance requirements of lactating Hereford cattle measured from 2- through 12 yr of age. To our knowledge, no other direct comparisons of maintenance energy requirements for young lactating vs. mature lactating beef cows are available. Currently, there is no age adjustment recommended for maintenance energy requirement of beef cows in the NASEM (2016) model and our results support this conclusion.

Estimates of maintenance requirements for young lactating cows in this experiment (94 kcal ME/kg BW^0.75^) are considerably lower than those reported for primiparous beef cows by Reynolds and Tyrrell (2000; 120 kcal ME/kg BW^0.75^) and Freetly et al. (2006a; 146 kcal ME/kg BW^0.75^). At least a portion of this difference could be explained by limit-feeding a high-energy diet. Freetly et al. (2006b) and Trubenbach et al. (2016) reported that limit-feeding high-energy diets to beef cows resulted in a reduction in energy required for maintenance. Furthermore, Freetly et al. (2006b) found that heat production rapidly declined through about 7 d after initiation of feed restriction and from d 28 to d 98, heat production continued to decline in a linear fashion. It is possible that potential differences in maintenance requirements due to age are diluted in the current experiment because 2- and 3-yr-old cows were comingled. For example, in a previous study using similar techniques and a similar diet, Wiseman et al. (2019) reported maintenance requirements of 107 kcal ME/kg BW^0.75^ in primiparous cows from the same herd.

Calf initial BW, final BW, and adjusted 205-d weaning BW were not affected by breed type. There was a tendency for lower daily feed energy intake in calves from HA-sired dams. This is the result of numerically lower initial calf BW and feed intake restriction at the rate of 1.25% of BW (DM basis). An explanation for improved gain per unit of feed energy intake in calves from HA-sired dams is not clear because there was no difference in BW gain per unit of total calf energy intake nor total pair energy intake.

The differences in genetic potential (i.e., milk production, growth, mature size, etc.) among breed types and their crossbred counterparts have been identified as a major source of variation in input requirements and efficiency of feed energy utilization (Ferrell and Jenkins, 1987; Solis et al., 1988; Jenkins et al., 1991). Although differences in dam and calf weight and energy intake were detected between breed types, Klosterman and Parker (1976), Marshall et al. (1976), Bowden (1981), and McMorris and Wilton (1986) found no differences in biological efficiencies between breeds and breed crosses when expressed as the energy requirement to produce a unit of weaning weight or pounds of retail product yield. Data from Holloway et al. (1985) and Montano-Bermudez and Nielsen (1990) suggest differences in efficiency were related to dam genetic potential for milk production and prioritization of nutrients to milk production over the lactation period. Although genomic scores reported for dams on study showed no differences in genetic potential for milk, RFI scores reported were lower (more efficient) for HA dams than ANG. This is in line with calf performance data from this study reporting calves from HA cows were more feed efficient than ANG calves. This agrees with Retallick et al. (2017), who found that Hereford heifers were more efficient in both ad libitum and restricted feeding environments when compared with Angus. Previous reports indicate that RFI is moderately heritable 0.40 to 0.52 (Arthur et al., 2001; Rolf et al., 2011). When evaluated as a pair, similarities between breeds in cow size, feed intake, milk production, and calf growth in the current study suggest that biological efficiencies between HA and ANG are not different when managed similarly.

Calves from young dams had lower initial BW, final BW, ADG, and adjusted 205-d weaning BW. As a result, feed energy intake was lower in calves from young dams because feed intake was restricted to 1.25% of current BW. Interestingly, calf gain per unit of calf feed energy intake was improved in calves from young dams. Young cows produced less milk than mature cows. Other researchers (Broesder et al., 1990; Abdelsamei et al., 2005) have found that as milk availability decreases, forage intake increases, but forage consumed to compensate for lower milk intake typically has a lower energy value than the milk it is replacing. However, because calves in the current study were limit-fed a high-energy ration, lower milk intake for calves from young dams may have resulted in improved feed energy utilization. Furthermore, data from Appleman and Owen (1975) showed that increased milk availability resulted in faster initial gains up to 84 to 112 d of age while Abdelsamei et al. (2005) found that milk availability over 8 kg/d decreased rate of increase in ADG. Overall, there were no differences in efficiency of calf gain per unit of calf total energy intake nor efficiency of calf gain per unit of pair total energy intake.

Despite differences in calf performance and calf energy intake, there were no differences between age groups in biological efficiency of calf growth. Jenkins et al. (1991) found slightly lower efficiency values for Angus/Hereford pairs at 35.8 g of calf gain per Mcal ME intake. The same study found that efficiency ratios were greater for Angus/Hereford pairs compared with other breeds of cattle that had a greater genetic propensity for growth and milk production. Similarly, Wiseman et al. (2019) found for traditionally weaned and early weaned primiparous Angus and Hereford × Angus pairs, efficiencies were 40.2 and 37.0 g of calf gain to total pair energy intake, respectively. It is important to note that both Jenkins et al. (1991) and Wiseman et al. (2019) offered ration ad libitum to calves while calf feed intake was restricted in the current experiment.

There was a tendency for greater BW in HA cows compared with ANG cows at the initiation of the voluntary forage intake experiment and this difference can be explained by increased BCS in HA cows. Voluntary forage intake in nonlactating, pregnant HA cows was 6% less than ANG cows when expressed as kg/d and 7% less when expressed as g/kg BW^0.75^. Overall, the crossbred cows in this experiment maintained a greater level of body condition and consumed less forage than ANG during gestation. These differences were independent of cow size and milk energy production. Across-breed comparisons for genetic variation in feed intake showed a propensity for Angus cattle to have a greater average daily feed intake compared with other breeds (Retallick et al., 2017). The same study found that, compared with Angus, Hereford heifers and steers consumed 788 and 962 g less per day, respectively. However, this difference in feed intake did not result in differences in ADG, suggesting potential differences in efficiency between the 2 breeds. In contrast, Crowley et al. (2010) found that while feed intake of Angus bulls was comparable with those of Hereford cattle, Hereford bulls were able to gain more efficiently at the same level of intake. Holloway et al. (1985) found that when fed the same level of nutrients, Angus × Hereford crossbred cattle preferentially partitioned nutrients toward maternal tissue as opposed to milk production. Increased BCS and lower voluntary forage intake in HA cows suggests improved ability to maintain body condition in environments or conditions where nutrients are scarce or limited. Furthermore, greater body condition of crossbred cows could result in improved reproductive performance (Richards et al., 1986; Houghton et al., 1990).

There was no difference in voluntary forage intake between young and mature cows when adjusted for differences in BW^0.75^. Furthermore, young cows were able to maintain similar body condition as mature cows and to compensate for slightly lower initial BW without differences in intake, suggesting forage utilization efficiency did not differ between age groups. Although young cows tended to have lower overall pregnancy rates (Table 4), a lack of differences in body condition throughout these studies coupled with no difference in maintenance requirements between age groups suggests that nutrient availability did not influence reproductive performance.

### Implications

For commercial producers, the largest economic benefit (66%) of crossbreeding comes from having crossbred cows (maternal heterosis; Weaber, 2015). Results from the current experiment suggest that Hereford genetics were complementary in a crossbreeding system with Angus cows to reduce cow/calf enterprise input costs. This advantage manifested as improved body condition and less ad libitum forage intake. The potential for reduced input costs need to be weighed with potential differences in productivity at the time of weaning and during the postweaning phases. Furthermore, younger cows have similar maintenance energy requirements and consume a similar amount of forage per unit of BW compared with mature cows.
